# Oxygen-controllable injectable hydrogel alleviates intervertebral disc degeneration by balancing extracellular matrix metabolism

**DOI:** 10.1016/j.mtbio.2024.101252

**Published:** 2024-09-14

**Authors:** Jia-Jie Lu, Qi-Chen Zhang, Guang-Cheng Yuan, Tai-Wei Zhang, Yu-Kai Huang, Tao Wu, Di-Han Su, Jian Dong, Li-Bo Jiang, Xi-Lei Li

**Affiliations:** a Department of Orthopaedic Surgery, Zhongshan Hospital, Fudan University, Shanghai, 200032, China; b Department of Orthopaedic Surgery, Zhongshan Hospital (Xiamen), Fudan University, Xiamen 361000, China; c Department of Orthopaedic Surgery, Shanghai Baoshan District Wusong Center Hospital, Fudan University, Shanghai, 200940, China

**Keywords:** Intervertebral disc degeneration, HIF-1α, Hypoxia-inducing hydrogel, ATI2341, Ultrasonic

## Abstract

Nucleus pulposus (NP) cells, situated at the core of intervertebral discs, have acclimated to a hypoxic environment, orchestrating the equilibrium of extracellular matrix metabolism (ECM) under the regulatory influence of hypoxia inducible factor-1α (HIF-1α). Neovascularization and increased oxygen content pose a threat, triggering ECM degradation and intervertebral disc degeneration (IVDD). To address this, our study devised an oxygen-controllable strategy, introducing laccase into an injectable and ultrasound-responsive gelatin/agarose hydrogel. Laccase-mediated reactions were employed to deplete oxygen, establishing a hypoxic microenvironment that upregulated HIF-1α expression. The activation of hypoxia-inducible factors significantly enhanced the expression of aggrecan and collagen II, concurrently suppressing Matrix metalloproteinases (MMP13) and A Disintegrin and Metalloproteinase with Thrombospondin motifs (ADAMTS5) levels, thereby restoring the equilibrium of ECM metabolism. Simultaneously, the hydrogel facilitated the recruitment of stem cells into the NP through the controlled release of ATI2341, activating C-X-C chemokine receptor type 4 (CXCR4). Moreover, ultrasound amplification enhanced ATI2341 release, promoting the migration of NP stem cells. The hydrogel's efficacy in mitigating metabolic imbalances and inhibiting IVDD progression was substantiated in a rat puncture IVDD model through hydrogel injection into the discs. In conclusion, this hypoxia-inducible hydrogel, responsive to thermal stimuli from ultrasound, presents a promising avenue for IVDD treatment.

## Graphical abstract

**Figure:**
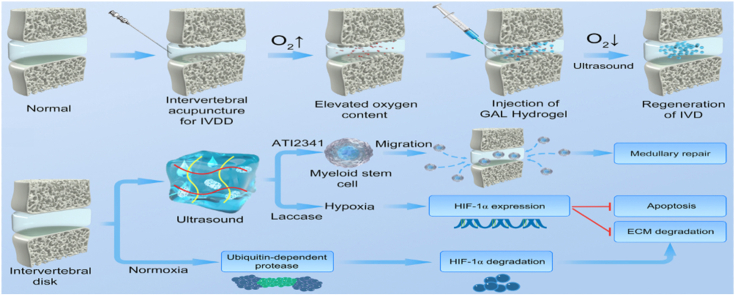

## 1 Introduction

IVDD has long been identified as a primary contributor to chronic low back pain [1,2]. Its characteristic features include abnormalities in the loading microenvironment, cellular dysfunction, and a disruption in the equilibrium of ECM synthesis and breakdown [3,4]. NP cells, existing within an avascular intervertebral disc, have acclimated to a low-oxygen microenvironment. However, the emergence of neovascularization elevates the oxygen content within the NP during IVDD. Excessive oxygen tension, in turn, fosters the expression of reactive oxygen species (ROS), culminating in disc degeneration [5]. Sequential passages conducted under hypoxic conditions underscore the importance of preserving a hypoxic microenvironment to sustain the phenotype of NP cells [6,7]. Consequently, maintaining this hypoxic microenvironment within the NP emerges as a potential and effective target for addressing disc degeneration [8].

HIF-1α, a hypoxia-sensitive transcription factor, initiates a sequence of cellular cascades, encompassing angiogenesis, cell proliferation, and metabolism, in response to a hypoxic environment [9]. It can activate autophagy through multiple pathways, regulating the homeostasis of various endogenous stem cells and ameliorating IVDD [10,11]. Intracellular HIF-1α activity is contingent upon oxygen levels. Under normoxic conditions, it undergoes degradation via the ubiquitin-dependent proteasome system, whereas in anoxic environments, its unhydroxylated state allows for stable maintenance. The pathophysiological state of disc degeneration induces alterations in the hypoxic environment, potentially contributing to the degradation of HIF-1α. Consequently, exploring whether hypoxia-induced HIF-1α expression holds therapeutic efficacy for IVDD becomes pertinent.

Reported hypoxia-inducible biomaterials can be categorized into two types based on their mechanisms: chemically induced and physically induced. The former, exemplified by the use of Dimethyloxalylglycine (DMOG) or Co_2_^+^, is predominantly employed for cartilage and bone repair [12,13]. The latter focuses on creating a physical hypoxic microenvironment by consuming oxygen, aiming to induce angiogenesis and investigate tumor metastasis under hypoxic conditions [14]. Notably, Sharon Gerecht et al. introduced a novel hypoxia-inducible hydrogel composed of gelatin and ferulic acid, presenting potential applications in treating hypoxic regulatory disorders [15]. This experimental design utilized laccase, a one-electron oxidoreductase found in various higher plants and fungi [16], to deplete oxygen. The mild reaction properties and biocompatibility of laccase made it a catalyst in redox reactions for diverse food processing applications [17]. In biological materials, it has been reported for reducing oxygen to water, thus establishing an anoxic microenvironment in the body [15]. However, its application in treating IVDD has not been explored. Consequently, our study devised a hydrogel capable of inducing a hypoxic microenvironment through the controlled release of laccase. This intervention aims to ameliorate IVDD by enhancing HIF-1α expression.

A thermosensitive hydrogel undergoes a phase transition in response to changes in the external temperature, making it an exceptional material for drug carriers and tissue engineering scaffolds [[18], [19], [20]]. Agarose, a polysaccharide derived from red algae, exhibits water solubility at 90 °C and forms a robust semisolid gel within a brief period as the temperature drops to 35–40 °C. This gel offers commendable biocompatibility, adjustable mechanical properties, and a modifiable water-absorbing capacity [21]. Gelatin, well-known for its biocompatibility, possesses the Arg-Gly-Asp (RGD) sequence that facilitates cell adhesion [22]. The sol-to-gel transition temperature for gelatin typically ranges from 22 to 31 °C. By combining these two gel solutions and adjusting the component ratios, the dissolution temperature of the hydrogel becomes flexibly controllable. This enables the construction of a temperature-sensitive hydrogel capable of solidifying at body temperature and dissolving at elevated temperatures [23]. Furthermore, this hydrogel demonstrates substantial degradability, rendering it practical for preventing inflammatory reactions arising from prolonged presence in the body [24,25].

The presence of stem cells within the NP introduces a novel concept in the treatment of IVDD, facilitating cell regeneration within this region [26]. The C-X-C motif chemokine ligand (CXCL12)-CXCR4 signaling pathway, recognized for inducing cell chemotaxis and proliferation, has been employed for NP repair [27]. However, the extended use of recombinant CXCL12 contained in hydrogels for prolonged treatment may lead to rapid degradation and inactivation. By contrast, ATI2341, an agonist of CXCR4, previously demonstrated its ability to induce chemotaxis in myeloid cells [28]. Consequently, utilizing ATI2341 to activate chemotaxis in stem cells presents itself as a promising option for treatment.

Ultrasound-induced cavitation and pyrolysis are pivotal in elevating local tissue temperature, facilitating enhanced fluid flow to promote material exchange, and even fostering cell growth [29]. Consequently, the development of hydrogel systems with ultrasound-responsive properties holds the promise of enhanced safety and efficacy in disease treatment [30,31].

**Scheme 1:**
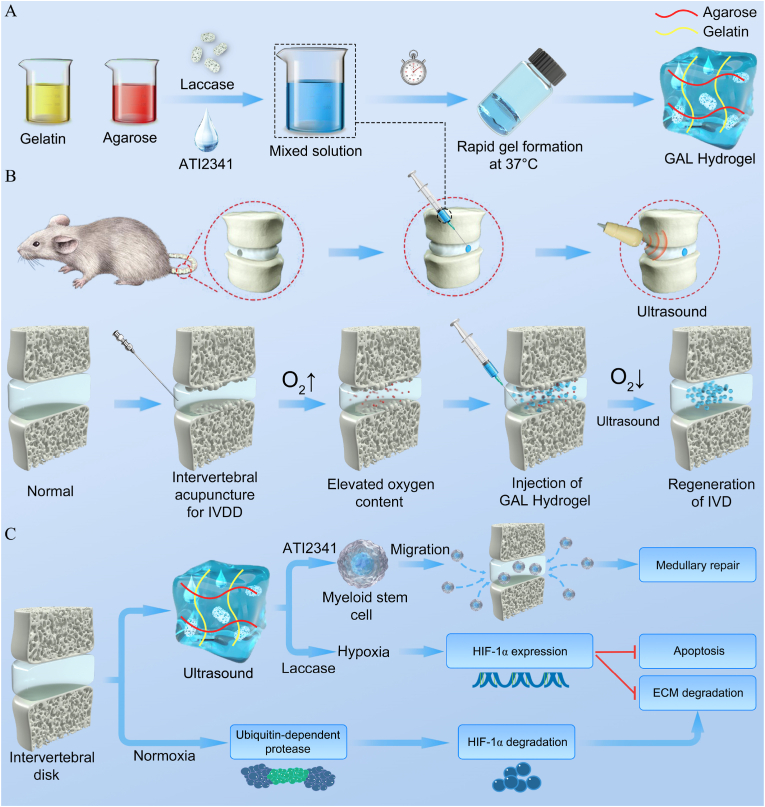
**Preparation scheme and therapeutic mechanism of Gelatin-Agarose-Laccase (GAL)-ultrasound responsive hydrogel.** (A) Construction of GAL hydrogels. (B) GAL hydrogel was injected into the intervertebral disc to induce a hypoxic microenvironment and synergized with ultrasound for intervertebral disc degeneration (IVDD) treatment. (C) Ultrasound-mediated improvement of IVDD by GAL hydrogel and the underlying mechanism.

In this study, an injectable ultrasound-responsive temperature-sensitive hydrogel consisting of laccase and ATI2341 was synthesized, where gelatin and agarose were used as carriers. In vitro experiments demonstrated that it effectively activated HIF-1α by inducing hypoxia thereby inhibiting NP cell apoptosis and alleviating ECM degradation. It also promoted NP cell growth under ultrasound and induced ATI2341 release stimulating NP stem cell migration. Eventually, by constructing a rat model of IVDD, it was confirmed that the hydrogel could promote intervertebral disc regeneration and ultimately achieve the purpose of intervertebral disc repair (Scheme 1).

## 2 Results and discussion

### 2.1 Preparation and characterization of GAL hydrogels

**Fig. 1:**
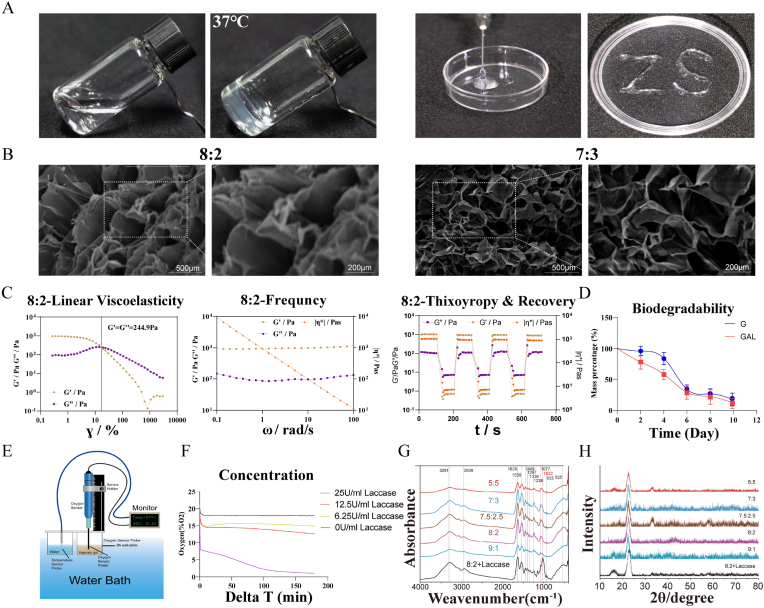
Characterization of hydrogels. (A) Physical and injection-ready properties of hydrogels. (B) Electron microscopic characterization and magnification of hydrogels (scale bar: 500 μm, 200 μm). (C) Rheological results of 8:2 hydrogel. (D) Biodegradability of GAL hydrogel. (E) Schematic of hydrogel oxygen content measurement. (F) Ability of different concentrations of laccase to induce hypoxic microenvironment in hydrogels. (G,H) FTIR and XRD of hydrogels of different ratios.

The compromised environment of a degenerated disc often results in the premature degradation or leakage of certain drugs and compounds before they can exert their therapeutic effects. Biomaterials offer a solution by encapsulating and releasing the drug or biologically active molecule under specific conditions, thereby optimizing performance [32,33]. Gelatin and agarose, renowned for their favorable gel properties, find extensive use in biomaterial preparation. Both solutions solidify into gels upon cooling, and by adjusting their proportions, a solid hydrogel can be achieved at approximately 37 °C. Maintaining the temperature at approximately 40–45 °C allows it to be stored as a solution in a syringe. Upon injection into the ambient temperature, approximately 37 °C (simulating body temperature), the hydrogel transforms from a sol to a gel (Fig. 1A). It takes about 1–2 min to gel (Fig. S2).

Scanning electron microscopy (SEM) images revealed a loose and porous structure of the hydrogel (Fig. 1B and Fig. S1 A), facilitating effective drug loading and cell growth. Strain scans and rheological characterization revealed that the storage modulus of the hydrogel in the solid state gradually decreased with an increasing gelatin fraction while the linear region shifted toward higher strains. In frequency scans, hydrogels with a 9:1 ratio at 37 °C displayed considerable variability in the dissipation modulus, deviating from the characteristics of an elastic system and indicating higher instability compared to other ratios. Thixotropy testing demonstrated the rapid transformation of hydrogels into a liquid state under 3000 % strain, recovering their solid properties after stress cessation. However, for the 9:1 gel fraction, the recovery ability after strain removal was lower than before (Fig. 1C and Fig. S1 C). In sterile PBS solution, the mass of GAL remained relatively stable for 4 days, with accelerated degradation observed from the fourth day onwards. Approximately 11 % of its mass remained undegraded after 10 days (Fig. 1D), indicating inherent biodegradability when maintaining a balanced ratio. This degradation rate establishes a stable microenvironment for laccase and ATI2341 in vivo during the initial days. As laccase and ATI2341 become functional, the hydrogel degrades, promoting HIF-1α expression, NP stem cell chemotaxis, tissue regeneration, and ultimately disc repair. The ability of laccase to induce a hypoxic microenvironment was tested using 8:2 hydrogels (Fig. 1E). Different concentrations of laccase induced hypoxic microenvironments at varying strengths and rates. A laccase concentration of 25U/mL effectively reduced the oxygen content of the hydrogel to less than 1 % (Fig. 1F). Maintaining the laccase concentration constant, the effect of laccase-induced hypoxic environments varied at different depths of the hydrogel (Fig. S1 B). Fourier-transform infrared (FTIR) spectroscopy showing an absorption peak at 1022 cm^−1^ for the hydrogel with 25 U/mL laccase. Conversely, hydrogels without laccase addition matched all characteristic peaks of gelatin and agarose (Fig. 1G). Moreover, the hydrogels were subjected to X-ray Diffraction (XRD) to observe whether they had specific structural alterations [34]. No change in the appearance of the indicated peaks in the hydrogel after the addition of laccase was observed after comparison (Fig. 1H).

### 2.2 Biocompatibility of hydrogels

**Fig. 2:**
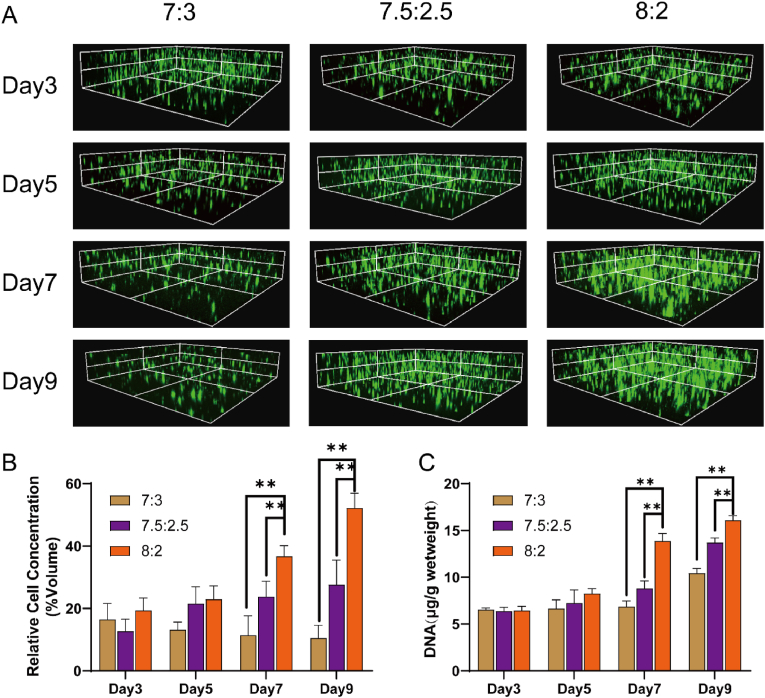
Biocompatibility and pressure testing of hydrogels. (A) Three-dimensional imaging of live–dead cell staining in hydrogels. (B) Statistical analysis of NP cell concentration in hydrogels. (C) DNA content in hydrogels. ∗∗*p* < 0.01, ∗*p* < 0.05, n = 3.

Biocompatibility is the foremost prerequisite for material applications. Building upon the observation that the hydrogel in the 9:1 group exhibited less stability at 37 °C and recognizing that gelatin contains more RGD sequences, favorable for the chemotactic growth of cells, efforts were directed toward cultivating cells in the 7:3, 7.5:2.5, and 8:2 groups. NP cells were introduced into the hydrogels, and live and dead cell staining was performed from 3 days post-inoculation. Three-dimensional reconstructions under a confocal microscope revealed notably superior cell growth in the 8:2 group compared to the other two groups (Fig. 2A and B). Additionally, DNA content within the hydrogel was assessed, revealing a significantly higher DNA content in the 8:2 group compared to the other two groups (Fig. 2C). This suggests that the 8:2 hydrogel provides a more conducive growth environment for cells.

### 2.3 RNA-Seq and bioinformatics analysis of various groups of NP cells

**Fig. 3:**
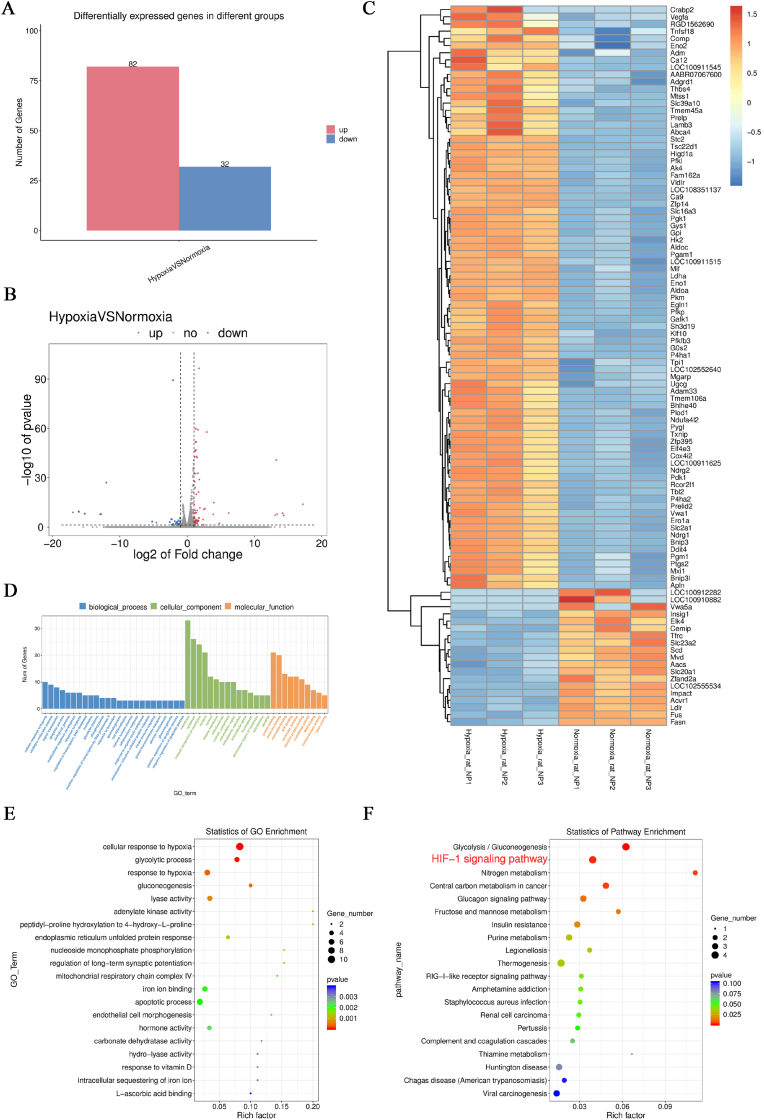
RNA-Seq and bioinformatics analysis results. (A) Number of differently expressed genes. (B) Volcano plots under normoxic and hypoxic conditions. Red clusters represent upregulated genes, and blue clusters represent downregulated genes. (C) Heatmap results: red indicates highly expressed genes, and blue indicates lowly expressed genes. (D,E) GO enrichment analysis. (F) KEGG enrichment analysis. (For interpretation of the references to colour in this figure legend, the reader is referred to the Web version of this article.)

RNA sequence analysis was conducted on NP cell groups, and gene expression was differentially analyzed using DESeq2. A total of 82 genes were significantly upregulated, while 32 genes were significantly downregulated in the hypoxic group compared to the normoxia group (Fig. 3A). Volcano and heat maps were generated to illustrate up- and downregulated genes through clustering analysis of differentially expressed genes (Fig. 3B and C). The heat map results indicated increased expression of genes closely related to or positioned upstream and downstream of HIF-1α, such as Vegfα, Comp, Adm, Ca12, Bnip3, Bnip3L, and Pdk1, in NP cells under hypoxic culture conditions. Differential genes underwent GO enrichment analysis at the biological process (BP), cellular component (CC), and molecular function (MF) levels. The results of GO enrichment analyses are presented with statistical significance at p < 0.05 (Fig. 3D and E). Additionally, KEGG enrichment analysis using ggplot2 presented scatter plots, revealing the top 20 KEGG pathways enriched in NP cells under hypoxic conditions. Notably, the HIF-1 signaling pathway emerged as statistically significant (p < 0.05) (Fig. 3F).

### 2.4 GAL induces hypoxia in vitro to promote HIF-1α expression

**Fig. 4:**
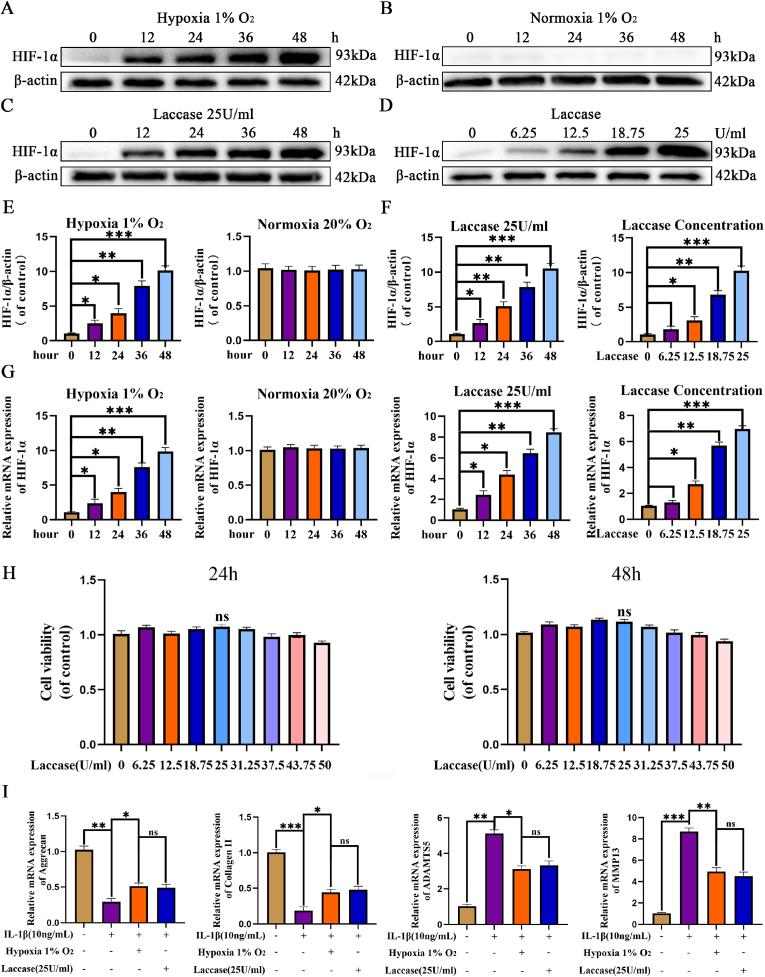
GAL promotes the expression of HIF-1α. (A–G) Western blot and PCR results of HIF-1α under different treatment conditions. (H) Cytotoxicity of laccase at varying concentrations on NP cells at 24 and 48 h. (I) PCR results of ECM expression under 1 % oxygen or laccase treatment. The data are shown as mean ± SD. ∗∗∗*p* < 0.001, ∗∗*p* < 0.01,∗*p* < 0.05, n = 3.

Hypoxia-induced activation of HIF-1α emerges as an effective therapeutic target for intervening in IVDD. In the context of intervertebral disc degeneration, the accelerated oxygen-dependent degradation of HIF-1α occurs owing to heightened oxygen concentration resulting from neovascularization and ROS production by senescent NP cells. Despite the traditionally perceived deleterious effects of hypoxia on NP cells, studies have shown its beneficial role in limiting ROS production and promoting cartilage matrix synthesis. Western blot results from this experiment illustrated an increase in HIF-1α expression over time in a hypoxic environment, contrasting with the opposite trend in normoxic conditions (Fig. 4A,B,E). Additionally, it was demonstrated that laccase could induce a hypoxic environment in the culture medium, promoting the expression of HIF-1α, with an optimal concentration observed at 25 U/mL (Fig. 4C,D,F). The qRT-PCR results were consistent with these findings (Fig. 4G). To assess laccase toxicity on NP cells, the Cell counting kit 8 (CCK-8 kit) was employed, and the results indicated that laccase had minimal toxicity after treating NP cells for 24 or 48 h (Fig. 4H).

### 2.5 GAL inhibits NP cell apoptosis and balances ECM metabolism in vitro

**Fig. 5:**
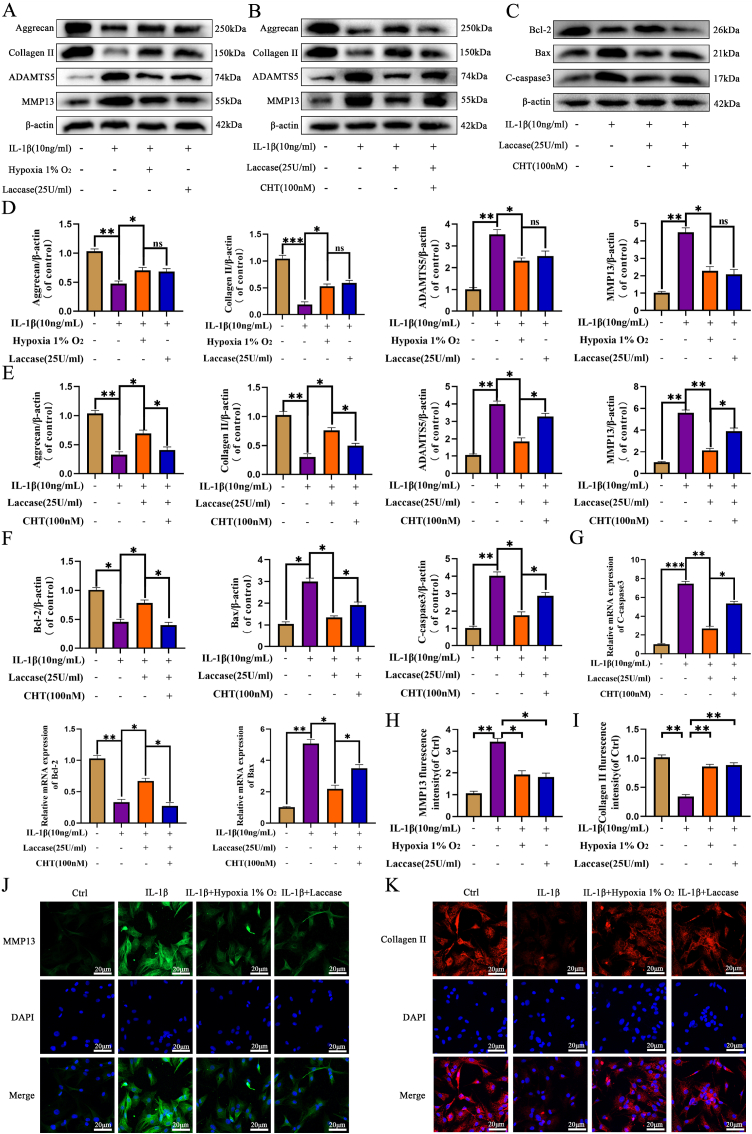
GAL inhibits IL-1β-induced apoptosis in NP cells and balances ECM metabolism. (A,D) Expression of ECM in IL-1β-induced NP cells treated with laccase and hypoxia 1 % O2. (B,C,E,F,G) Detection of ECM expression and degree of apoptosis under HIF-1α inhibitor chetomin treatment conditions using western blot and PCR. (H–K) Collagen II (scale bar: 20 μm) and MMP13 (scale bar: 20 μm) levels were detected using immunofluorescence combined with DAPI staining for NP cells. The data are shown as mean ± SD, ∗∗∗*p* < 0.001, ∗∗*p* < 0.01, ∗*p* < 0.05, n = 3.

IVDD is closely related to ECM metabolic homeostasis. To explore whether HIF-1α expression could affect the metabolic homeostasis of ECM, the study examined ECM expression under 1 % oxygen and laccase treatment using qRT-PCR (Fig. 4I) and western blot (Fig. 5A–D). Results indicated that 1 % oxygen and laccase could reverse the degradation of aggrecan and collagen II, while suppressing the expression of ADAMTS5 and MMP13 under IL-1β treatment. Immunofluorescence results of MMP13 and Collagen II further demonstrated the protective effect of hypoxia on ECM (Fig. 5H–K), affirming that laccase effectively creates an oxygen-deficient environment. To investigate whether the ameliorative effect on IVDD in a hypoxic environment is mediated by HIF-1α, chetomin (CHT, 100 nM) was employed to block HIF-1α activity by binding to EP300 (E1A-binding protein p300). Western blot and qRT-PCR outcomes (Fig. 5B,C,E,F,G) indicated that in the presence of chetomin, the effect of laccase was diminished, confirming that the alleviation of IVDD through HIF-1α activation in a hypoxic environment is mediated by HIF-1α. In addition, we used IL-1β(10 ng/mL) to induce intervertebral disc degeneration. The extracellular matrix of degenerative nucleus pulposus cells showed a reduction in aggrecan、collagen II and an increase in MMP13. The RNA expression of aggrecan, collagen II, MMP13 in nucleus pulposus cells was detected by q-PCR. The results showed that the expression of aggcrecan and collagen II in the nucleus pulposus was significantly up-regulated in GALU. The RNA expression of the extracellular matrix in the GA and GAL group was also increased to some extent when compared with the IL-1β group, and the elevation of MMP13 caused by IL-1β stimulation was also reversed by the GALU group (Fig. S4 A). The protein expression of aggrecan, collagen II and MMP13 in nucleus pulposus cells was detected by Western blotting (Fig. S4 B). The expression of extracellular matrix aggcrecan and collagen II in the nucleus pulposus was significantly upregulated by GALU. They were also increased in GA and GAL group when compared with the IL-1β group. The IL-1β-induced elevation of MMP13 was reversed under treatment in the GALU group, and the GA and GAL groups also exhibited repair of MMP13. Furthermore, the cellular immunofluorescence results also demonstrated the reparative effect of GALU on IVDD (Fig. S4 C).

### 2.6 GAL promotes NP stem cell chemotaxis in vitro

**Fig. 6:**
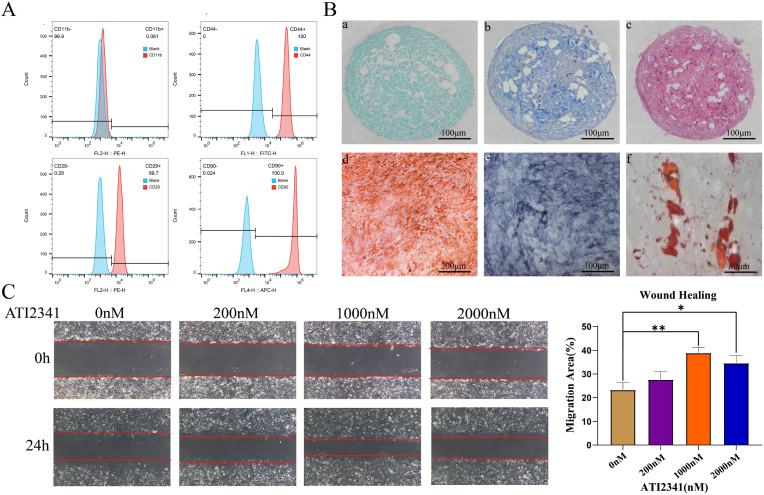
ATI2341-induced NP stem cell chemotaxis. (A) Flow cytometry results after staining rat NP cells with CD11b-PE, CD44-FI, CD29-PE, and CD90-AlexaFlour 647. (B) (a–f) are alisin blue, toluidine blue, SO, alizarin red, ALP, and oil red O staining of chondrospheres formed by induced rat NP cells, respectively. (C) Scratch experiments on NP cells using different concentrations of ATI2341. (For interpretation of the references to colour in this figure legend, the reader is referred to the Web version of this article.)

To perform NP repair, we confirm the presence of stem cells in the NP and characterize the extracted cells, surface markers such as CD44, CD29, CD90 (stem cell markers), and CD11b (myeloid-derived cell marker) were identified using flow cytometry (Fig. 6A). The results indicated that the cells extracted and cultured in this experiment exhibited characteristics consistent with the surface markers of stem cells. Subsequently, rat NP cells were subjected to differentiation and staining in three lineages. NP cells formed cartilage spheres in chondrogenic differentiation and were successfully stained with Alcian Blue, Toluidine Blue, and SO staining. Osteogenic differentiation revealed the formation of calcium crystals, successfully stained with Alizarin Red and ALP staining. In adipogenic differentiation, NP cells formed lipid droplets and stained positively for oil red O (Fig. 6B). This confirmed the presence of a substantial number of stem cells in the NP, forming the basis for implantation of materials for NP repairment. Considering the role of CXCL12-CXCR4 signaling pathway in inducing cell chemotaxis and proliferation and repairing the NP, CXCL12 has been used to treat intervertebral disc degeneration and has shown good efficacy [26]. Previous studies have shown that CXCL12 induces stem cell chemotaxis and promotes proliferation by activating the ERK pathway [35,36]. However, evidence reveals that CXCL12 expression is elevated in the serum of patients with intervertebral disc degeneration and induces apoptosis of NP cells through the NF-κB signaling pathway [37]. Furthermore, recent studies have shown that ATI2341 plays an important role as an agonist peptide of CXCR4 [28,38]. Therefore, this study uses ATI2341 as a substitute for CXCL12 for subsequent experiments. A scratch assay was performed to evaluate the chemotaxis effect of ATI2341 on NP cells. Results indicated that at appropriate concentrations, ATI2341 effectively promoted NP stem cell chemotaxis, showing a statistically significant effect at 1000 nM, although the chemotaxis induced by ATI2341 tended to decrease with a further increase in dosage (Fig. 6C).

### 2.7 GAL response and cell growth-promoting ability under ultrasound action

**Fig. 7:**
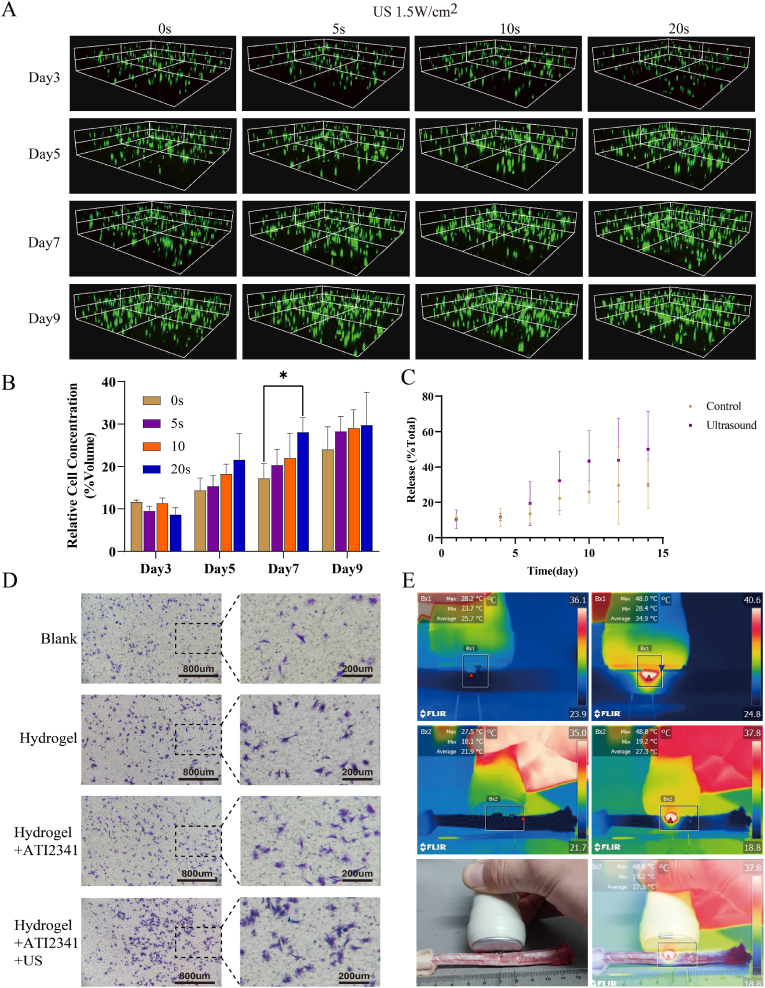
Effect of ultrasound on GAL hydrogels and NP cells. (A,B) Hydrogels containing nucleus pulposus cells were stained with live and dead cells for 3D imaging and statistical analysis under different ultrasonic durations. (C) Drug release curve of hydrogels treated by ultrasound. (D) Transwell assay on NP cells using extracts. (E) Infrared imaging of rat caudal intervertebral discs irradiated with ultrasound. The data are shown as mean ± SD, ∗∗*p* < 0.01, ∗*p* < 0.05, n = 3.

The potential impact of the thermal and cavitation effects of sonication on cell viability was considered in this study, particularly within the hydrogel. Cells were inoculated into hydrogels from day 3 onwards, with sonication applied every other day. Results indicated a significantly higher number of viable cells in the 20 s sonication group compared to other groups at day 7 (Fig. 7A and B). While more cells were counted at other time points with longer sonication durations, statistical significance was lacking. This demonstrated that ultrasound, under appropriately controlled conditions, can promote NP cell growth to a certain extent. Additionally, ultrasound may have stimulating effects on intervertebral disc cells similar to those observed in the NP cells, potentially contributing to improved disc repair [[39], [40], [41]]. As a drug-carrying hydrogel, drug release from the hydrogel was evaluated in this experiment. The concentration of the drug in the hydrogel extract was measured using High Performance Liquid Chromatography (HPLC). Results indicated an elevated mean concentration from day 6 onwards in the ultrasound group compared to the control group, with the rate of elevation decreasing after exceeding 40 % of the total amount(Fig. 7C). Gel precipitation observed after lyophilization of the extracts and subsequent re-dissolution indicated enhanced fluid exchange induced by sonication, promoting drug release from the hydrogel. Given the superior chemotaxis effect of ATI2341 at 1000 nM, this concentration was used to encapsulate the hydrogel. Ultrasound was then applied for 30 s on days 2 and 4 of gel formation, with the extract collected on day 5. Transwell experiments on NP cells using this extract demonstrated better chemotaxis of NP cells (Fig. 7D). Using external heating devices such as hot packs is not enough to cause deep tissue heating, and low-frequency ultrasound penetrates the deep tissue to achieve adequate heating effect [42]. Moreover, among all organs in the human body, bones exhibit the strongest absorption capacity for ultrasound. The intervertebral disc is located between the upper and lower vertebrae, and to detect whether the energy is absorbed by the vertebrae or transferred to other vertebrae, this study explored the ability of ultrasound to produce local heat in the vertebrae using infrared imaging. Results indicated that ultrasound could quickly and accurately heat local tissues after local coupling drops, with adjacent vertebrae remaining unaffected (Fig. 7E). This finding is crucial as bones have a strong absorption capacity for ultrasound, and the localized warming of bone tissue can contribute to increased hydrogel responsiveness to ultrasound within the disc, providing additional benefits.

### 2.8 GALU attenuates a puncture model of intervertebral disc degeneration in rats

**Fig. 8:**
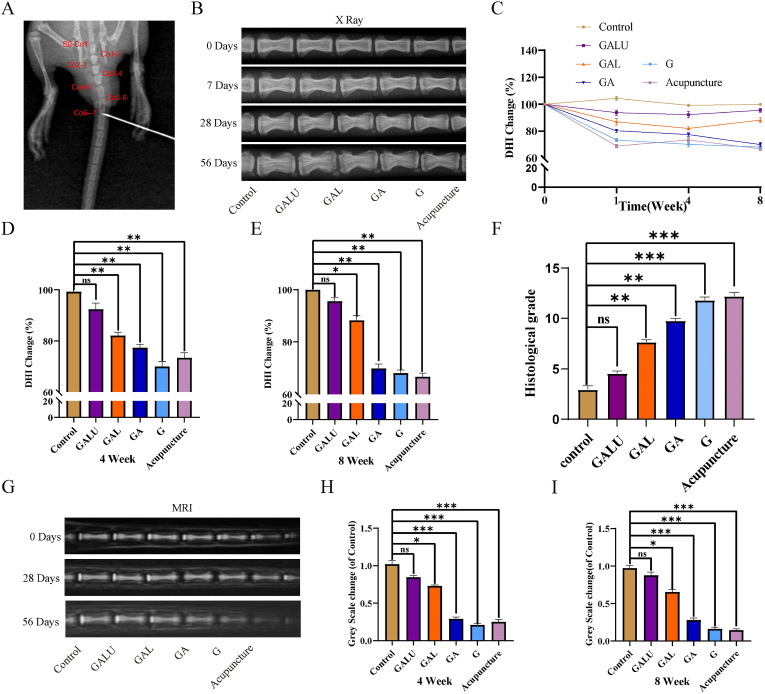
Imaging of GALU hydrogel-repaired rat intervertebral disk. (A) Rat caudal spine acupuncture model. (B) X-ray imaging of the rat tail spine. (C–E) Line and bar graph analysis of the rat tail spine DHI change at different times. (F) Histologic grading of different groups at 8 weeks post-operation. (G) MRI images of the rat caudal spine. (H,I) Gray scale values of rat caudal intervertebral discs at 4 and 8 weeks. The data are shown as mean ± SD, ∗∗∗*p* < 0.001, ∗∗*p* < 0.01, ∗*p* < 0.05, n = 3.

In the final evaluation, the therapeutic efficacy of the injectable hypoxic hydrogel, which is thermally responsive to ultrasound, was tested in a rat puncture model of intervertebral disc degeneration. A rat model of intervertebral disc degeneration was established with the assistance of a C-arm machine (Fig. 8A). Hydrogel was injected immediately after modeling, and based on the hydrogel composition, the groups were divided into a puncture group (Acupuncture), a hydrogel-alone group (G), a hydrogel + ATI2341 group (GA), a hydrogel + ATI2341+laccase group (GAL), and a subgroup of the GAL group where rats underwent ultrasound treatment every 3 days (GALU). Intervertebral disc height (DHI) was used as an indicator reflecting changes in the extracellular matrix (ECM [43]). The results revealed that the DHI of rats in the GALU group was not significantly different from that of the control group at weeks 4 and 8. The GAL group showed a certain degree of improvement in DHI with conditional significance, albeit not as effective as the GALU group. The efficacy of the GA and G groups was not significant, similar to the acupuncture-only group, suggesting a better therapeutic effect of the GALU group on IVDD (Fig. 8B–E). Histologic scores were calculated according to previous reports [44]. In the GALU group, the histologic scores decreased significantly at postoperative week 8 and were not significantly different from those of the control group, indicating repair of disc degeneration. In the GAL, GA, and G groups, histologic scores increased over time and were statistically significant, indicating an ongoing disc degeneration process that was not significantly inhibited (Fig. 8F). Magnetic resonance imaging (MRI) can reflect IVDD water content, with higher gray levels being associated with lower water content [45]. The MRI results at postoperative weeks 4 and 8 showed that the gray value of the GALU group was smaller than that of the control group, though not statistically significant. The GAL group had a less effective result compared to the GALU group, while the water content of intervertebral discs in the GA and G groups significantly decreased, similar to the acupuncture-alone group (Fig. 8G–I).

**Fig. 9:**
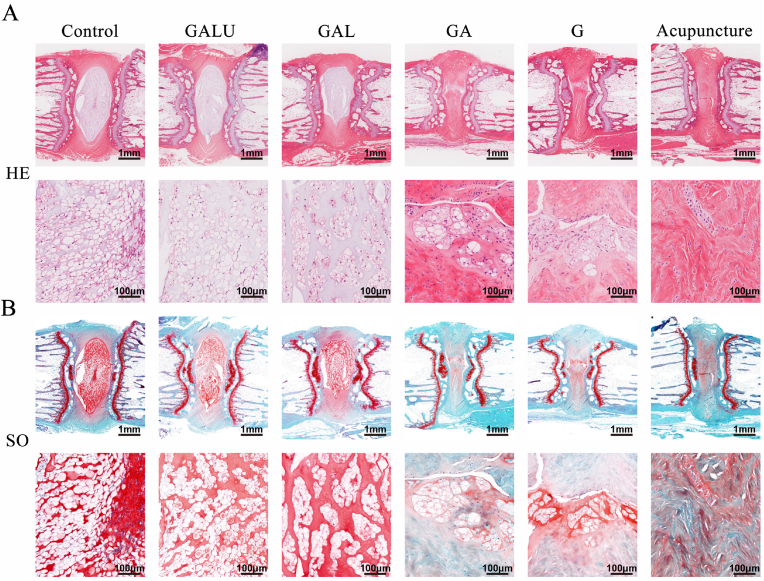
Histological analysis of GALU hydrogel repair of NP in rats. (A,B) H&E and SO staining of rat intervertebral disc sections after 8 weeks of modeling. The data are shown as mean ± SD, ∗∗∗*p* < 0.001, ∗∗*p* < 0.01, ∗*p* < 0.05, n = 3.

Histological sections of the NP at postoperative week 8, subjected to Hematoxylin and eosin (H&E) and Safranin O staining, revealed that the GALU and GAL groups had clearer NP and fibrous ring boundaries, with the NP more completely preserved. The GALU group showed better results than the GAL group. By contrast, the GA and G groups exhibited blurred NP and fibrous ring borders, greater damage to the NP, and poorer treatment outcomes (Fig. 9A and B).

## 3 Conclusion

In summary, this study devised an injectable hypoxia-inducible hydrogel comprising gelatin, agarose, and laccase in a ratio conducive to gel formation at body temperature. The hydrogel effectively induced a hypoxic microenvironment, activating HIF-1α upon laccase incorporation. Additionally, it induced the migration of NP cells upon mixing with ATI2341, and ultrasound irradiation further promoted NP cell migration and cell growth within the hydrogel. Consequently, the application of this hydrogel in conjunction with ultrasound treatment demonstrated efficacy in repairing disc degeneration induced by needle modeling in the rat caudal spine. This approach presents a novel strategy for the biotherapy of disc degeneration.

## CRediT authorship contribution statement

**Jia-Jie Lu:** Investigation, Data curation. **Qi-Chen Zhang:** Software, Investigation, Formal analysis. **Guang-Cheng Yuan:** Software, Project administration, Data curation. **Tai-Wei Zhang:** Project administration, Investigation. **Yu-Kai Huang:** Formal analysis, Data curation, Conceptualization. **Tao Wu:** Data curation, Conceptualization. **Di-Han Su:** Formal analysis, Data curation, Conceptualization. **Jian Dong:** Methodology, Investigation, Funding acquisition. **Li-Bo Jiang:** Writing – review & editing, Writing – original draft, Supervision, Investigation, Funding acquisition. **Xi-Lei Li:** Writing – review & editing, Supervision, Funding acquisition.

## Declaration of competing interest

The authors declare that they have no known competing financial interests or personal relationships that could have appeared to influence the work reported in this paper.

## Data Availability

Data will be made available on request.
