# Supplementary material for: Oxygen-controllable injectable hydrogel alleviates intervertebral disc degeneration by balancing extracellular matrix metabolism
Source: Mater Today Bio. 2024 Sep 14;29:101252. doi: 10.1016/j.mtbio.2024.101252 (PMC11609683; doi:10.1016/j.mtbio.2024.101252)
Supplement: Multimedia component 1 [file mmc1.docx]

**Oxygen-controllable injectable hydrogel alleviates intervertebral disc degeneration by balancing extracellular matrix metabolism**

*Jia-Jie Lu, Qi-Chen Zhang, Guang-Cheng Yuan, Tai-Wei Zhang, Yu-Kai Huang, Tao Wu, Di-Han Su, Jian Dong*, Li-Bo Jiang*, Xi-Lei Li**

J.-J. Lu, Q.-C. Zhang, G.-C. Yuan, T.-W. Zhang, Y.-K. Huang, T. Wu, D.-H. Su, J. Dong*, L.-B. Jiang*, X.-L. Li *

Department of Orthopaedic Surgery

Zhongshan Hospital

Fudan University

Shanghai 200032, China

1. mail: li.xilei@zs-hospital.sh.cn; jiang.libo@zs-hospital.sh.cn; dong.jian@zs-hospitcal.sh.cn.

J. Dong*

Department of Orthopaedic Surgery

Shanghai Baoshan District Wusong Center Hospital

Fudan University

Shanghai 200940, China

**KEYWORDS**: Intervertebral disc degeneration, HIF1-α, Hypoxia-inducing hydrogel, ATI2341, Ultrasonic.

1. **Materials and Methods**

**1.1 Ethics statement**

The experimental design was approved by the Animal Protection Committee of Zhongshan Hospital, Fudan University. The "Guidelines for the Care and Use of Laboratory Animals" issued by the National Institutes of Health of the United States of America is an important document for the handling and use of animals in this experiment. We purchased 4-week-old male SD rats of SPF grade from Shanghai Jieshijie Laboratory Animal Center and housed them in the Experimental Research Center of Zhongshan Hospital, Fudan University.

**1.2 Materials and Reagents**

Ethylenediaminetetraacetic acid (EDTA) and gelatin were obtained from Aladdin Biochemical Technology Co (Shanghai, China). Agarose was obtained from Biowest (Loire Valley, France). Laccase and CCK-8 kit were bought from Yuan Ye BiotechnologyCo (Shanghai, China). ATI2341 was purchased from MCE (New Jersey, USA). The Ultrasound coupling agent and IL-1β were obtained from Tsuya Technology Development Co (tianjin,China). 5×Protein Free Closure Solution and PBS were acquired through Enzyme Biotechnology Co (Shanghai, China). Dimethyl sulfoxide (DMSO) and Alcian Blue 8GX were bought from Sigma Aldrich (Shanghai, China). QuickBlock™ Western and CIP/NBT alkaline phosphatase colorimetric kit were bought from Biyun Tian Biotechnology Co (Shanghai, China). (HRP)-goat anti-mouse/ rabbit secondary antibody were acquired through JacksonImmunoResearch (Pennsylvania, USA). ECL Enhanced Chemiluminescence Liquid was acquired from Yisheng Biotechnology Co (Shanghai, China).Toluidine Blue Stain was bought from Saiwei technology Co (Wuhan, China). Dulbecco's modified Eagle's medium (DEME) and Penicillin Streptomycin were acquired through Hyclone (Utah, USA). TrypLE™ Express and chetomin were acquired through Gibco (NY, USA). Collagenase P was bought from Roche (Shanghai, China). DAPI was bought from Bold Bioengineering Co (Wuhan, China). Buffered glycerol、Citric acid buffer and FBS were bought from Sangong Bioengineering Co (Shanghai, China). Stem Cell Culture Kit were purchased from Sai Ye Biotechnology Co (Shanghai, China). Live-Dead Cell Staining Kit and DNA Quantitation Kit were purchased from KGI Biotech Co (Jiangsu, China). Primary antibodies Bax, Bcl-2, C-caspase3 and β-actin were purchased from Cell Signaling Technology (Beverly, USA). Primary antibodies ADAMTS5, Aggrecan, HIF-1α, collagen II and MMP13 were bought from Abcam (Cambridge, MA, USA).

**1.3 Preparation of Gelatin-Agarose-Laccase hydrogels**

Solution A: 600 mg of gelatin was added to 5 ml of double-distilled water, 60℃ water bath, inverted and stirred to dissolve the solid completely. Solution B: Add 10 ml of double-distilled water to 200 mg of agarose, remove from microwave oven when heated to boiling and shake well, repeat several times until no visible solid particles. Solution C: Laccase was dissolved in double-distilled water and filtered through a 0.22 μm filter head. ATI2341 was dissolved in DMSO at a concentration of 1 mM. Solutions A, B, and C were mixed in the proportions shown below, then ATI2341 was added and placed at 37℃ to obtain the desired hydrogel (Table S1).

**1.4 Characterization of GAL hydrogels**

The GAL hydrogels were quenched in liquid nitrogen and then placed in a lyophilizer for freeze-drying. The completed lyophilized samples were affixed to a copper sample stage using conductive adhesive, sprayed with gold and placed in a Hitachi S-4800 SEM system for observation. Subsequently, these samples were ground to a non-granular powder and potassium bromide powder was added for further grinding, and then placed in a Nicolet FTIR 6700 spectrometer for structure scanning. XRD was performed in a Rigaku Ultima IV and scanned over a range of 10-80° at a speed of 10°/min. Rheologics of the GAL hydrogel was performed on the HAAKE MARS 60 rotational rheometer, and the test temperature was set at 37 °C. For the strain scanning, the frequency was fixed at 1 rad/s and the strain range was 0.3-3000%; and for the frequency scanning , the frequency range was 100-0.1 rad/s and the strain was fixed at 1%; and for the thixotropy scanning, the large strain was 3000% at the time of gel disruption for 60s, and the strain was 1% at the time of monitoring the recovery of the gel structure afterwards.

**1.5 Ultrasonic on the GAL hydrogels**

The hydrogel without cells was placed in a 1.5 ml centrifuge tube, and after applying a coupler on the ultrasound probe the centrifuge tube was placed on its side in the coupler, and ultrasound transmission was performed for 1.5 minutes at 1.5 W/cm^2^. The cell-loaded hydrogel was inoculated in a 24-well plate. Target wells were placed on the coupling agent and sonicated at 1.5 W/cm^2^. For animals, rats were immobilized in a restrictor every 3 days, and after applying a small amount of couplant to the puncture site, ultrasound was performed at 1.5 W/cm^2^ moving in a circular pattern around the rat's tail for 1 minute at a time and labeled.

**1.6 HPLC**

1uM of ATI2341 was diluted 5-fold for three consecutive times and analyzed using a SCIEX QTRAP 6500+ LC-MS/MS system to obtain the characteristic ion peaks and establish a standard curve. A 500μl 8:2 hydrogel containing 1 uM ATI2341 was placed in a 1.5ml centrifuge tube, 500μl of double-distilled water was added and placed in a 37°C thermostat. The hydrogels were divided into two groups. For the hydrogel in the ultrasound group, daily ultrasound interventions were performed from day 3 onwards. 500μl of leachate was collected after daily sonication intervention and frozen in -80°C refrigerator for a total of 16 days. The extracts were lyophilized and resuspended in 200μl of double-distilled water, analyzed using a SCIEX QTRAP 6500+ LC-MS/MS system and calculated.

**1.7 Rat intervertebral disc degeneration model**

We purchased 8-week-old male SD rats of SPF grade from Shanghai Jieshijie Laboratory Animal Center for in vivo experiments. The rats were anesthetized with 3% pentobarbital at a dose of 40 mg/kg by intraperitoneal injection. After positioning under the C-arm machine, the rats were punctured from Co6-7 to Co10-11 using an 18G needle, and the needle was inserted into the intervertebral disc and rotated 180 degrees after a sensation of falling out was felt. A 1ml syringe with a 25G needle was then used to inject 50μl of pre-prepared hydrogel placed in a water bath at approximately 40°C into the model intervertebral disc. Specimens were collected at 8 weeks postoperatively. A total of 32 rats were used for in vivo experiments, with 8 rats in each group, to prevent accidental death of rats in the course of feeding and reduce the reliability of the experiment. Samples were fixed with 4% formamint at 4°C for 48h and decalcified with 10% EDTA solution for 2 months at 4°C. The samples were then dehydrated with graded ethanol solutions. Sections were sectioned after paraffin embedding. The sections were then stained with H&E and SO.

**1.8 Extraction and culture of NP cells from SD rats**

To obtain rat NP Cells, 4-week-old SD rats were sacrificed, and NP tissue was isolated using micro instruments. Collagenase P (Roche, Basel, Switzerland, 11249002001) and TrypLE™ Express (Gibco, 12605028) were used to digest the tissue at 37°C for 4 h and 30 min. NP cells were then seeded in DMEM containing 10% FBS (Gibco, 10099-141C) and antibiotics (1% penicillin/streptomycin), and cultured at 37°C in a humidified incubator with 95% air and 5% CO2 (generation 0). When the cells reached 80–90% confluence, they were detached with 1×TrypLE Express Enzyme and subcultured in culture flasks. Generation 2 NP cells were used in all the experiments.

**1.9 NP stem cell characterization and Flow cytometry**

The flow antibody was added to 4 groups of centrifuge tubes containing NP cells, gently blown and mixed and then placed in the refrigerator at 4℃ for 15 minutes of incubation away from light; 500μl of PBS was added and centrifuged for 5 minutes, and the supernatant was discarded; 200μl of PBS was then added, resuspended and transferred to the flow tubes, and fluorescence intensities of the cells in each group were detected in the C6 PLUS flow cytometer.

**1.10 Chondrogenic induction and phenotyping**

Add all dexamethasone, ascorbic acid, ITS additive, sodium pyruvate and proline provided in the Saiye Rat Bone Marrow Mesenchymal Stem Cells into Chondrocyte-induced Differentiation Medium Kit into the SD Rat Bone Marrow Mesenchymal Stem Cells into Chondrocyte-induced Differentiation Basal Medium and mix completely to make a l premix, and then aspirate 5 μl of TGF-β3 provided in the kit into 0.5 ml of the premix every 3 days, and add this was used as the complete culture medium for chondrospheres. The NP cells were centrifuged in a centrifuge tube for 5 min, supernatants were removed, cells were resuspended using 0.5 ml of premix and centrifuged again for 5 min. The supernatant was removed and the cells were resuspended with 0.5 ml complete medium and centrifuged for 5 min. Subsequently, the cells were incubated in an incubator at 37°C, 5% CO2. Fluid changes were performed every 3 days using 0.5 ml complete medium. Chondrospheres were harvested on days 21 and 28, fixed using 4% paraformaldehyde, embedded in paraffin, and finally identified. Embedded sections of cartilage sphere specimens fixed in 4% paraformaldehyde were commissioned to Wuhan Xavier Biotechnology Co. Sections were baked at 60℃ and placed in a thermostat overnight. On the next day, the sections were deparaffinized by immersion in xylene for 15 min each time in two batches. The sections were dehydrated with 100%, 95%, 90%, and 75% alcohol and placed in each concentration for 5 minutes. Finally, the sections were washed with double-distilled water and various stains were identified.

**1.11 Osteogenic induction and ALP and alizarin red staining**

Osteogenic induction solution was prepared using the Sayer Rat Bone Marrow Mesenchymal Stem Cells Osteogenic Induction and Differentiation Medium Kit. Briefly, add all serum, double antibody, glutamine, dexamethasone, ascorbic acid, and sodium β-glycerophosphate provided in the kit to the SD rat bone marrow mesenchymal stem cell osteogenic induction and differentiation basal medium and mix completely, which is called complete medium. Cells from the culture dish were digested down and inoculated equally in 6-well plates using TrypLE™ Express. Osteogenic induction was performed when the cell density reached approximately 70%-80%. The original medium was removed and 2 ml of complete medium was added to each well, changed every three days, and after 4 weeks the cells were fixed using 4% paraformaldehyde and finally stained with ALP and alizarin red.

**1.12 Lipogenic induction and oil red O staining**

Prepare lipid-induced differentiation medium for rat bone marrow mesenchymal stem cells using Sayer's Rat Bone Marrow Mesenchymal Stem Cells Lipid-induced Differentiation Medium Kit. The 20 ml serum, 2 ml double antibody, 2 ml glutamine, 200 μl dexamethasone, 400 μl insulin, 200 μl 3-isobutyl-1-methylxanthine, and 200 μl rosiglitazone provided in the kit were added to the SD rat bone marrow mesenchymal stem cells lipid-induced differentiation basal medium and mixed completely, which was referred to as A solution. Add another 20 ml of serum, 2 ml of double antibody, 2 ml of glutamine, 400 μl of insulin, 2 all provided in the kit to the basal medium of SD rat bone marrow mesenchymal stem cells into lipid-induced differentiation and mix completely, called solution B. Cells were digested from 10c dishes using TrypLE™ Express and inoculated into six-well plates. After overnight incubation the cells were adherent to the wall and spread over the bottom of the wells, at this point lipidogenic induction could be started. The original medium was removed and 2 ml of solution A was added to each well, which was replaced by 2 ml of solution B after three days and then by solution A after 24 hours, and so on five times. The cells were continuously cultured with liquid B for 6 days, and the liquid was changed every 3 days. After 6 days, the cells were fixed using 4% paraformaldehyde and stained with Oil Red O.

**1.13 Three-dimensional culture of NP cells**

Cells were digested down in Petri dishes with TrypLE™ Express, counted using a DeNovix CellDrop BF Cell Counter, placed in centrifuge tubes and centrifuged for 5 min, after which cells were resuspended using freshly prepared 400 μl of sterile hydrogel placed in a water bath at 40°C, and the hydrogel cells were inoculated into 24-well plates and spread well. 500 μl of phenol red free high sugar medium containing 15% FBS was added to each well and cultured in an incubator at 37°C containing 5% CO2, and the fluid was changed every other day.

**1.14 Staining of live-dead cells**

Live-dead cell staining was performed using the KGI Live-Dead Cell Staining Kit. Calcium xanthophyll and PI were mixed with PBS at the ratio of 5μl/10ml of PBS for both, and the live-dead cell staining solution was added after aspirating the medium from the 24-well plate, and then returned to the incubator and incubated at 37°C for 15min, and then the staining solution was aspirated after observing fluorescence staining under a fluorescence microscope, and then changed back to phenol red-free high sugar medium containing 15% FBS, and photographed and reconstructed in three dimensions under a confocal microscope.

**1.15 X-ray radiographs in small animals**

Small animal X-rays were taken using an MX-20 soft X-ray radiograph. Rats were anesthetized with 1% pentobarbital at a dose of 40mg/kg by intraperitoneal injection, placed in lateral recumbency, with their tails taped to the radiographs, straightened and slightly fixed, placed in the X-ray machine, the site of the shot aligned under the laser, and a metal pin placed around the body markings for easy identification under the X-rays. Samples were photographed using automated parameters. The samples were analyzed for intervertebral space height index (DHI Changes) in ImageJ. The specific method is to divide the endplates on both sides of the disc into four equal parts, and the intervertebral space heights measured at the 1/4, 1/2, and 3/4 equal points are recorded as a, b, and c. Then the lengths of the three equal points of this disc to the caudal endplates of the discs on the cephalad side and the cephalad endplates of the discs on the caudal side of the vertebrae are measured at the three equal points and are recorded as d, e, f, and g, h, and i, respectively; and the DHI is 2(a+b+c)/(d+e+f+g+h+i), and the change in DHI is postoperative DHI/preoperative DHI×100%.

**1.16 Magnetic resonance in small animals**

Magnetic resonance images of rat caudal intervertebral discs were taken using a SIEMENS Verio 3.0T magnetic resonance system. Briefly, 1% pentobarbital was anesthetized in rats by intraperitoneal injection at a dose of 40mg/kg, and the tails of the rats were straightened and pasted on a foam plate placed in a knee coil, and photographed using the t2_tse_cor sequence with a set resolution of 384×384 and a layer thickness of 1.5mm. The grayscale values of each intervertebral disc were analyzed using Weasis software after photographing.

**1.17 Hydrogel Oxygen Consumption Test**

Suspend the 96-well plate in a water bath and adjust the water temperature to 37°C. 100μl of hydrogel mixed with laccase was rapidly added to the wells of the 96-well plate, and the change in oxygen content was continuously monitored.

**1.18 Alcian blue staining**

Drops of Alisin blue working solution onto the slide, incubate for 30 minutes at room temperature, rinse with water until no stain remains, dry and seal the slide with neutral resin and observe the staining results.

**1.19 Toluidine blue staining**

Toluidine blue dye solution (0.1%) was added dropwise on the slide, which was then incubated at room temperature for 10min, washed with distilled water three times, 5min each time, dried, and mounted with neutral gum to visualize the staining results.

**1.20 Immunofluorescence staining**

Cells were washed with 0.5% Triton-100 for 30min, blocked with protein-free rapid blocking solution diluted in PBST for 10min, and then incubated with Collage II and MMP13 primary antibodies (1:200 dilution) at 4°C overnight. Cells were subsequently washed three times with PBS for 5min each, Cy3-labeled goat anti-rabbit secondary antibody was added, and washed after incubation for 1h in a dark environment. The cells were stained with DAPI staining solution for 20min and mounted, and finally observed under a fluorescence microscope.

**1.21 RNA extraction together with qRT-PCR analysis**

Total cellular RNA was isolated with TRIzol reagent (Invitrogen), followed by reverse transcription of the isolated RNA for the preparation of cDNA using the cDNA Reverse-Transcription Kit (Invitrogen/Life Technologies). Syber Green real-time reverse transcription polymerase chain reaction (RT-PCR) technique was used to evaluate the expression of genes. In brief, the reaction was carried out at a total volume of 10µL, which consisted of 5µL Syber Green, 0.25µL primers, and 4.5µL cDNA. Gene expression data were analyzed based on the 2−ΔΔCt method with β-actin as a housekeeping gene.

**1.22 NP cells RNA-Seq and Bioinformatics Analysis**

Two samples were selected from Hypoxia-rat-NP group and Normoxia-rat-NP group respectively for RNA-seq analysis, which was repeated three times and subjected to RNA extraction, detection, enrichment of mRNA, PCR amplification, and library detection, etc. Sequencing, data quality control, and quantification of gene expression levels were performed by Shanghai Lianchuan Biotechnology Co. Cutadapt software was used to remove the reads that contained adaptor contamination. And After removed the low quality bases and undetermined bases ,used HISAT2 software to map reads to the genome. The mapped reads of each sample were assembled using StringTie with default parameters. Then, all transcriptomes from all samples were merged to reconstruct a comprehensive transcriptome using gffcompare software. After the final transcriptome was generated, StringTie and ballgown were used to estimate the expression levels of all transcripts and perform expression level for mRNAs by calculating FPKM. The differentially expressed mRNAs were selected with fold change > 2 or fold change < 0.5 and p value < 0.05 by R package edgeR or DESeq2, and then analysis GO enrichment and KEGG enrichment to the differentially expressed mRNAs.

**1.23 Hydrogel DNA content detection**

Measurement of hydrogel DNA content was performed using DNA Quantitation Kit, Fluorescence Assay. Briefly, hydrogels containing 100μg/ml DNA and 1mg/ml DNA were prepared by adding calf thymus DNA in different ratios. The hydrogels containing cells and standard DNA concentration were well broken and mixed using an ultrasonic cell breaker. Add 0, 2, 5, 10ul of hydrogel containing 100μg/ml DNA and 2, 5, 10μl of hydrogel containing 1mg/ml DNA and 10μl of cell-loaded hydrogel to each well of 96-well plate. 200μl of 2mg/ml Hoechst 33258 was added to each well under light-avoiding conditions, shaken for 3min in an enzyme labeling apparatus and then irradiated using a 360nm laser and the intensity of excitation light at 460nm was collected. The DNA standard curves for different ratios of hydrogels were calculated and the DNA content of each sample was calculated.

**1.24 Western blotting**

A western blot assay was performed to determine protein levels. The cell pellets were lysed in RIPA buffer to extract the total protein. In brief, after the addition of RIPA buffer, the cells were kept on ice for 15min, then sonicated and centrifuged at 12,000 RPM for 30min at 40C. Total protein was calculated with a bicinchoninic acid (BCA) protein detection kit (Beyotime, Shanghai, China). Proteins were separated through sodium dodecyl sulfate-polyacrylamide gel electrophoresis (SDS-PAGE) and transferred to a polyvinylidene fluoride (PVDF) membrane (Bio-Rad, California, USA). Subsequently, the membrane was blocked with 5% bovine serum albumin (Beyotime, Shanghai, China) at 37°C for 2h with gentle agitation, followed by probing with primary antibody overnight at 4°C in the refrigerator. The membrane was then probed with secondary antibodies of corresponding molecular weight and incubated for 2h at 37°C. The membrane was washed three times with TBTS and visualized using enhanced chemiluminescence (MeiluneBio). In addition, NP cells were co-cultured with different groups of hydrogels for 24 hours and protein expression levels were measured by the WB method described above. The images were acquired using the Image Lab 3.0 software (Bio-Rad).

**1.25 Wound healing test**

The NP cells were evenly inoculated in 6-well plates and cultured until the bottom of the wells were spread, a straight line of uniform thickness was drawn through the cells at the bottom of the wells with a 200μl gun tip, the dish was shaken to suspend the cells that had been drawn through, and the medium and suspended cells were aspirated. New medium mixed with different concentrations of drug was added, photographed immediately, and the same area was photographed again 24h later. Different groups of hydrogels were added to the medium to co-culture with NP cells and experiments were performed as described above. The area of the scratched area was analyzed on imageJ and the degree of healing was calculated.

**1.26 Transwell experiment**

The hydrogels were soaked with equal volumes of high sugar medium containing 15% FBS, sonicated on days 3 and 5, and the culture medium from day 5 was collected. 600μl of day 5 medium was placed in the lower chamber of the Transwell, and 5000 cells were resuspended using 100μl of high sugar medium containing 15% FBS and then inoculated in the upper chamber of the Transwell. 24h later, the cells were fixed using 4% PFA for 15min. The chambers were stained in crystal violet solution for 15min. Subsequently, the cells at the bottom of the upper chamber were gently wiped away using a cotton swab, and the cells on the Transwell membrane were observed under a microscope and counted.

**1.27 Measurement of oxygen concentration in rat intervertebral discs**

We anesthetized rats and used a calibrated Presens micro-oxygen probe to puncture the corresponding intervertebral discs, and after confirming the proper puncture site under the C-arm machine, we rotated out the sensor probes and measured the oxygen content of the discs at different time periods (Fig S5).

**1.28 Statistical data analysis**

GraphPad Prism 8 software was used for all statistical analyses of data in this study, and the data results of quantitative information were expressed as mean±standard deviation, student-t test was used for two-group comparison, One way analysis of variance (ANOVA) was used for more than two-group comparison, and Bonferroni method was used for two-by-two comparison between groups. p<0.05 was considered statistically different.


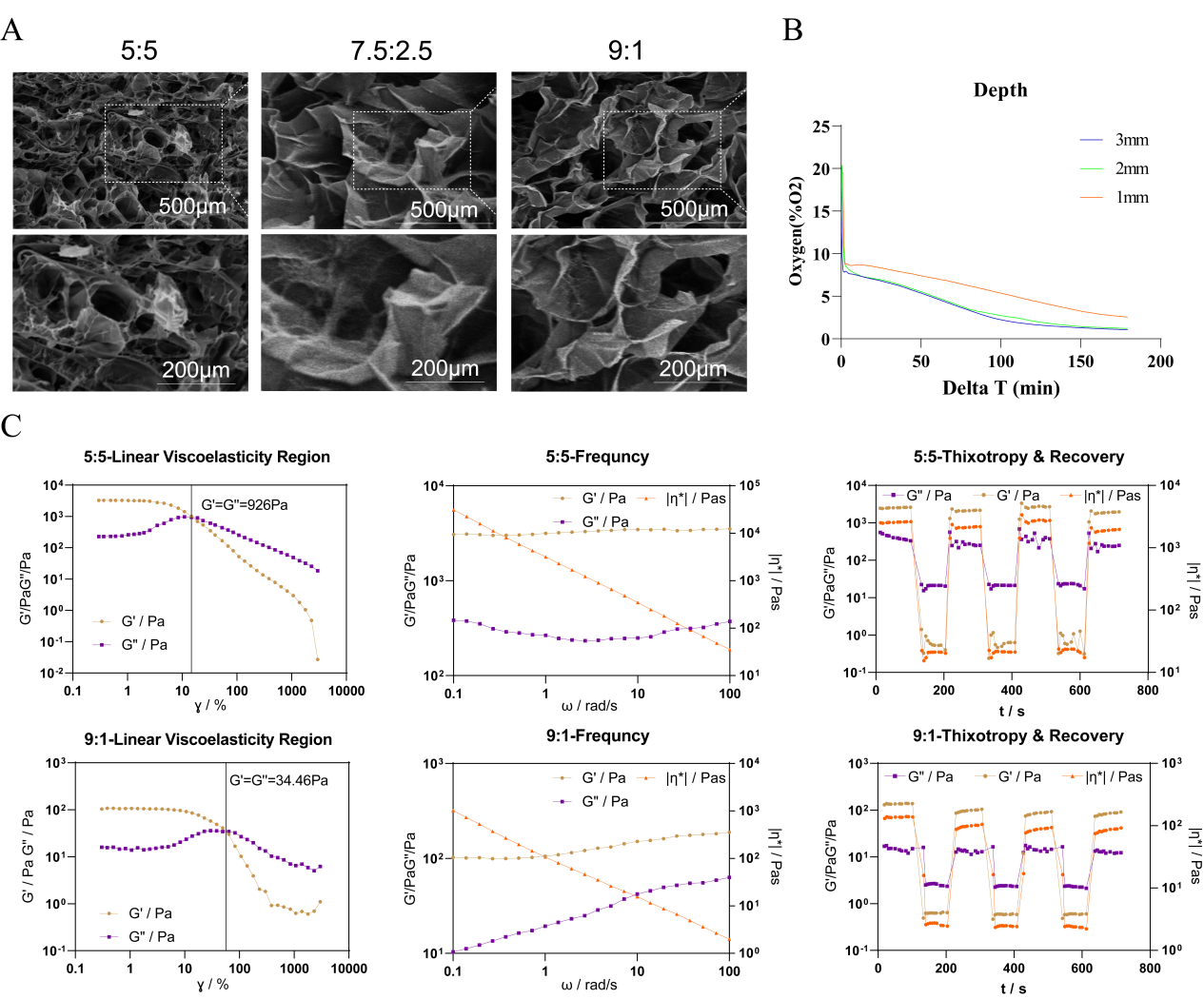


**Fig S1. Characterization of hydrogels.** (A) Electron microscopic characterization and magnification of hydrogels (scale bar: 500 µm, 200 µm). (B) The ability of laccase to induce hypoxia at different depths. (C) Rheological results of 5:5 and 9:1 hydrogels.


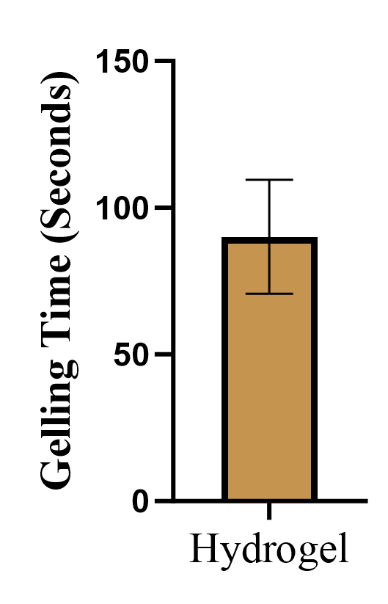


**Fig S2. Hydrogel gelling time.**

**
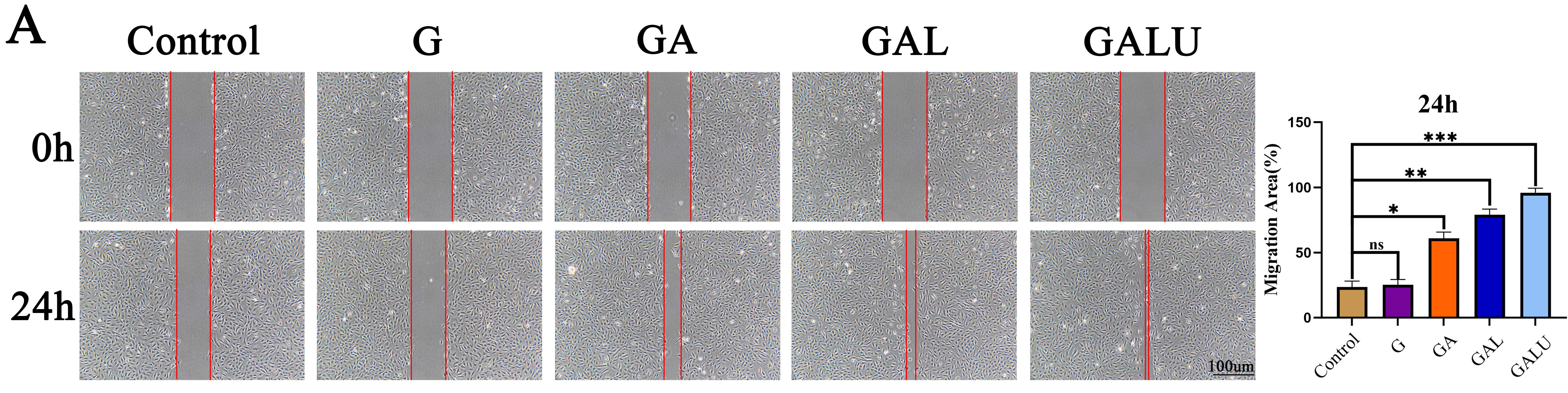
**

**Fig S3. Scratching test.** (A) Promotion of cell migration after 24 hours of co-culture of hydrogels with NP cells under different conditions (scale bar: 100 µm). The data are shown as mean ±SD, ***p < 0.001, **p < 0.01, *p < 0.05, n = 3.


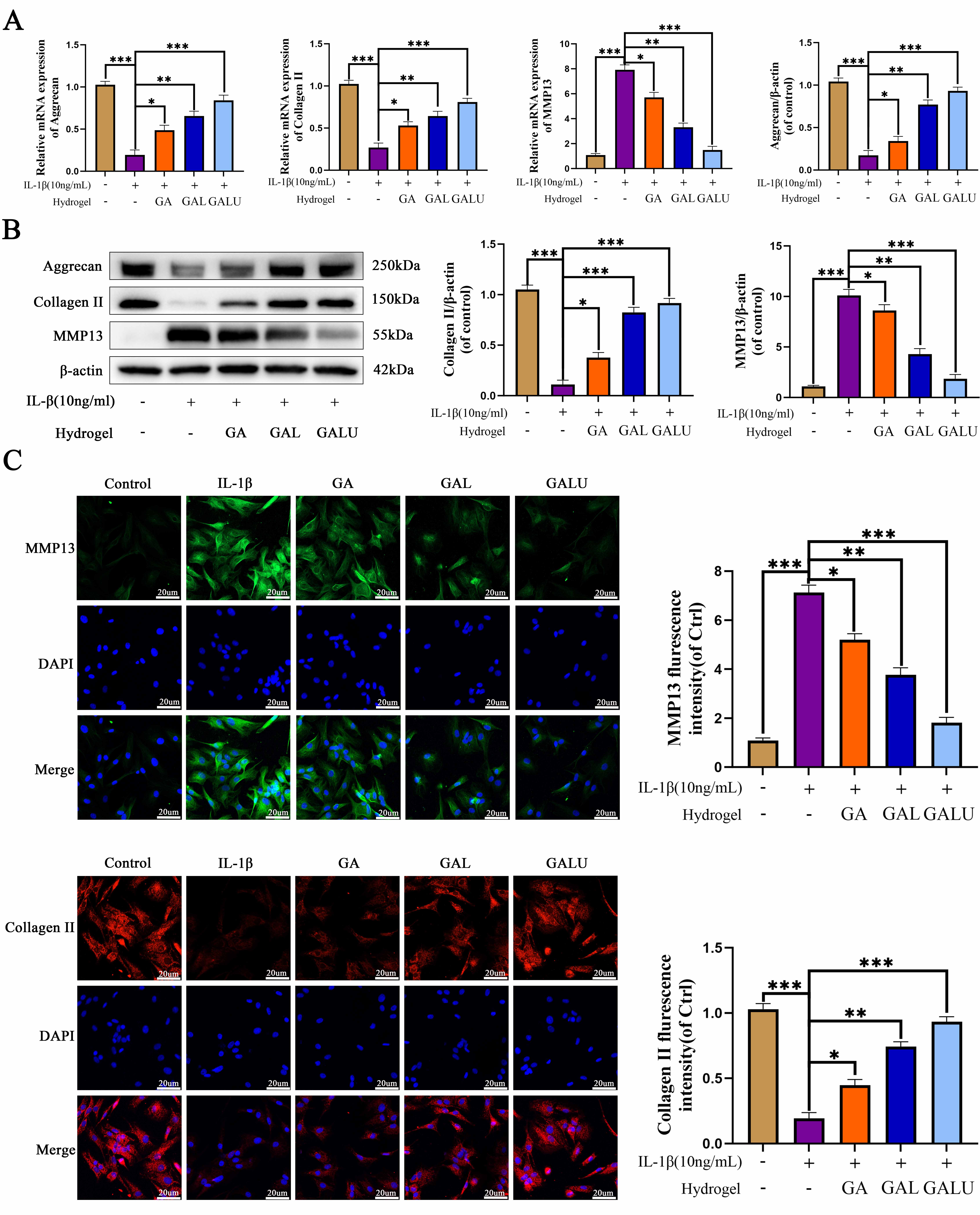


**Fig S4. Biological function of GALU in vitro cell experiment.** (A) PCR expression results of ECM treated with GA, GAL and GALU. (B) Western blot results showed that GA, GAL, and GALU treatments reversed IL-1β-induced changes in ECM. (C) Collagen II (scale bar: 20 µm) and MMP13 (scale bar: 20 µm) levels were detected using immunofluorescence combined with DAPI staining for NP cells. The data are shown as mean ±SD, ***p < 0.001, **p < 0.01, *p < 0.05, n = 3.


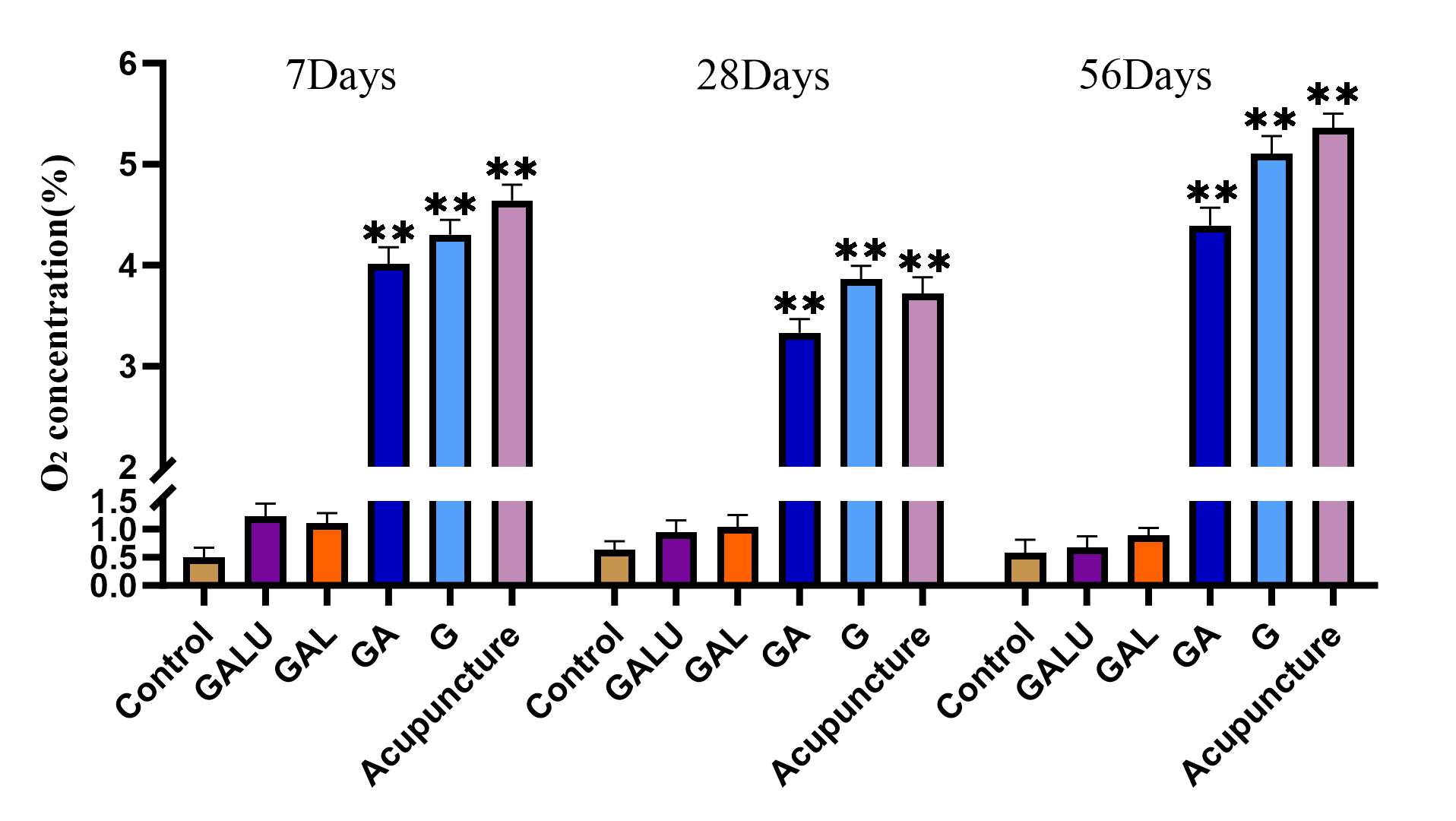


**Fig S5. Measurement of oxygen concentration in rat intervertebral discs.** The data are shown as mean ±SD, **p < 0.01, *p < 0.05, n = 3.


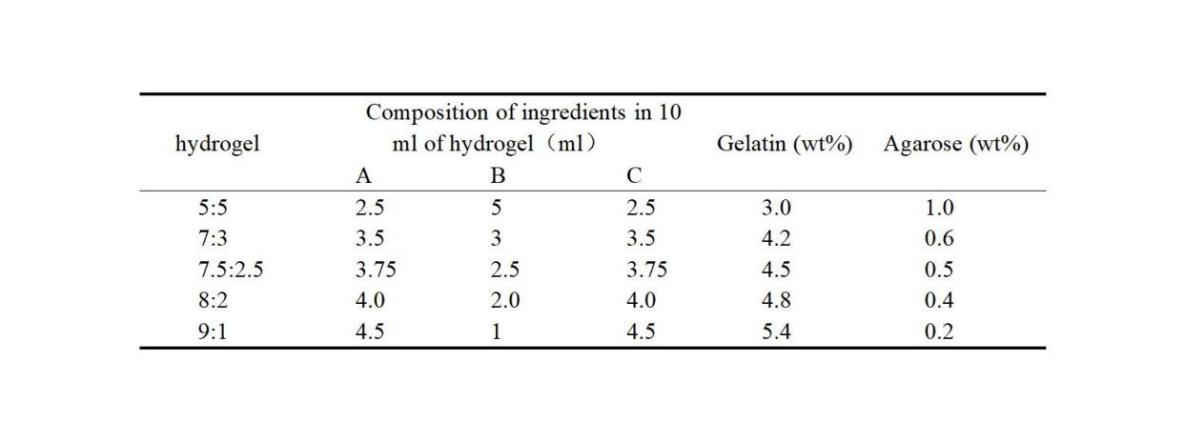


**Table S1. Preparation of hydrogels with different ratios.**


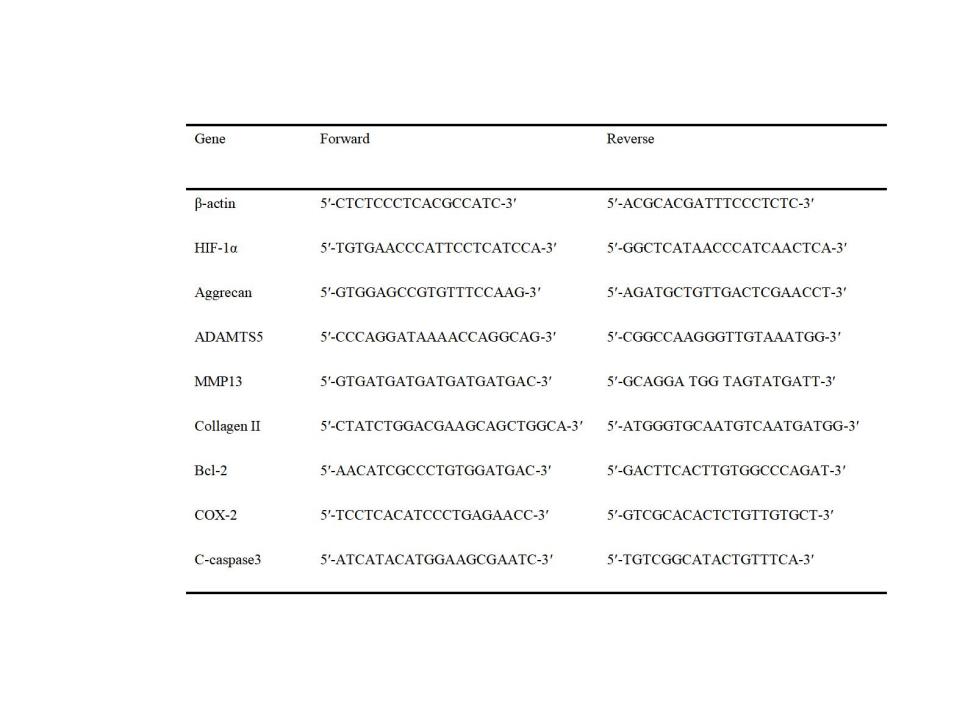


**Table S2. Primers used in real-time PCR.**
